# Bone marrow adipoq-lineage cells in regulating the bone and marrow microenvironment: identities, functions, and mechanisms

**DOI:** 10.3389/fendo.2026.1922395

**Published:** 2026-08-13

**Authors:** Chunyan Zhang, Xiaohui Gu, Yuyan Xiang, Chanchan Zheng, Lei Yang, Jianquan Chen

**Affiliations:** 1 Department of Clinical Medicine, School of Medicine, Hangzhou City University, Hangzhou, Zhejiang, China; 2 Center for Rehabilitation Medicine, Department of Orthopedics, Zhejiang Provincial People’s Hospital (Affiliated People’s Hospital, Hangzhou Medical College), Hangzhou, Zhejiang, China; 3 Center for Health Sciences and Engineering (CHSE), Hebei Key Laboratory of Biomaterials and Smart Theranostics, School of Health Sciences and Biomedical Engineering, Hebei University of Technology, Tianjin, China

**Keywords:** adipo-lineage progenitors, adipo-osteoprogenitors, adipoq-lineage cells, bone marrow niche, hematopoiesis, osteoclastogenesis, skeletal stem and progenitor cells

## Abstract

Bone marrow Adipoq-lineage cells refer to all cells that have ever expressed Adipoq (Adiponectin) and their progeny. They contribute not only to marrow adipogenesis but also to osteoclast regulation, hematopoietic support, vascular maintenance, and post-injury regeneration. Recent single-cell transcriptomic and genetic fate-mapping studies have revealed unexpected heterogeneity within this lineage, such as genuine adipogenic precursors (MALPs), bipotent adipo-osteoprogenitors (Adipoq^+^Osx^+^ cells), and their differentiated progeny (including mature marrow adipocytes and, in some contexts, osteoblasts). The progenitor subsets partly overlap with well-characterized SSPC subsets such as CXCL12-abundant reticular cells and leptin receptor-expressing cells, yet they exhibit distinct functional properties. This review synthesizes current knowledge on the identity, differentiation potential, and multifaceted functions of bone marrow Adipoq-lineage cells, with a focus on their roles in promoting osteoclastogenesis, regulating osteogenesis, supporting hematopoiesis and vascular integrity, and mediating bone marrow repair after injury. We also discuss how these cells are regulated by intrinsic factors that either control cell-autonomous fate or modulate secretory phenotype, and highlight key unresolved questions. By integrating these findings into a unified conceptual framework, this review aims to guide future research and therapeutic development targeting these cells in skeletal and hematopoietic disorders.

## 1 Introduction

The postnatal skeleton harbors heterogeneous populations of skeletal stem and progenitor cells (SSPCs) that are essential for bone growth, maintenance, and repair (1–4). Among these, adiponectin (also known as Adipoq)-expressing stromal cells (Adipoq^+^ stromal cells), have attracted considerable interest. Initially considered as a specific marker of mature adipocytes, Adipoq is now known to be expressed in some immature stromal cells that have diverse physiological functions (5, 6). Specifically, single cell RNA-sequencing (scRNA-seq) studies have identified a mesenchymal cell cluster highly expressing *Adipoq* with little expression of osteogenic markers, referred to as marrow adipogenic lineage precursors (MALPs) or Adipo-CAR cells (7, 8). These cells mainly occupy perivascular and reticular stromal spaces, form a reticular network throughout the marrow, and give rise to Perilipin^+^ lipid-laden adipocytes under steady-state conditions (5, 6). Importantly, recent work identified another subset of Adipoq^+^ stomal cells that are Adipoq^+^Osx^+^ and predominantly reside in the endosteal niche and function as bipotent adipo-osteoprogenitors in adult mice (9). These two populations represent distinct subsets of Adipoq-lineage progenitors. The broader category of bone marrow Adipoq-lineage cells encompasses all cells that have ever expressed Adipoq and their progeny, as fate-mapped by constitutive *Adipoq-Cre* or inducible *Adipoq-CreER^T^²*. This category includes Adipoq-lineage progenitors (e.g., MALPs and Adipoq^+^Osx^+^ cells) and their differentiated progeny (including mature marrow adipocytes and, in some contexts, osteoblasts), all of which profoundly influence bone mass, marrow adiposity, hematopoiesis, and vascular regeneration (5, 6). Notably, *Adipoq-Cre* labels progressively more osteoblasts with age. The recent identification of Adipoq^+^Osx^+^ bipotent progenitors provides a cellular basis for this observation in trabecular bone; whether additional mechanisms contribute remains to be determined.

Despite these advances, the identity and differentiation potential of bone marrow Adipoq-lineage cells remain subjects of active debate. Early studies using constitutive *Adipoq-Cre* drivers suggested that these cells are unipotent adipocyte precursors with little or no capacity for osteoblast differentiation (7, 10). By contrast, subsequent work using inducible lineage tracing demonstrated that the endosteal subset of Adipoq-lineage cells actively contributes to both osteoblasts and adipocytes even under steady-state conditions (9). These seemingly contradictory findings are now thought to reflect heterogeneity within the Adipoq-lineage, differences in skeletal sites, and the potentially progressive off-target labeling of osteoprogenitors by *Adipoq-Cre* with age (5, 6).

This review critically examines the identity, functions, and regulatory mechanisms of bone marrow Adipoq-lineage cells, placing them within the broader context of SSPC biology as defined in recent comprehensive reviews (1–3). The key terms introduced above, such as Adipoq^+^ stromal cells, Adipoq-lineage progenitors, MALPs, Adipoq^+^Osx^+^ cells, and Adipoq-lineage cells, are systematically summarized in **Table 1**. However, we should acknowledge that all currently available genetic tool (either constitutive or inducible *Adipoq-Cre*) label the entire Adipoq-lineage, and cannot selectively targets Adipoq^+^ stromal cells or MALPs. Consequently, functional studies using these *Cre* tools reflect manipulation of the whole lineage. We therefore attribute phenotypes to the Adipoq-lineage unless intersectional or scRNA-seq evidence supports a narrower assignment.

**Table 1:** Key Adipoq-related cell populations in the bone marrow.

Cell population	Key markers	Anatomical localization	Differentiation potential	Relationship to other populations
Adipoq−Lineage Cells (Broadest Definition)	Ever expressed Adipoq; labeled by *Adipoq−Cre* or *Adipoq−CreER^T^²*	Perivascular, stromal, endosteal, adipocyte compartments	Heterogeneous: includes adipo-progenitors, adipo osteoprogenitors, potentially bona fide Adipoq^+^ SSPCs, and their terminally differentiated progeny	encompassing Adipoq-lineage progenitors (including MALPs and Adipoq^+^Osx^+^ cells) and their differentiated progeny (including mature marrow adipocytes and, in some contexts, osteoblasts).
Adipoq^+^ Cells	Currently express *Adipoq* (detected by reporter, antibody, or scRNA-seq); includes Adip progenitors and mature adipocytes	Perivascular, stromal, endosteal, and adipocyte compartments	Heterogeneous: includes lineage-restricted (MALPs), bipotent (Adipoq^+^Osx^+^), potentially multipotent (Adipoq^+^ SSPCs) and terminal differentiated (adipocytes) subsets	Subset of Adipoq-lineage cells; includes MALPs, Adipoq^+^Osx^+^ cells, Adipoq^+^ SSPCs, and mature adipocytes
Adipoq^+^ Stromal Cells	Currently express Adipoq; negative for mature adipocyte markers (Perilipin^-^, no lipid droplets)	Perivascular and reticular stromal spaces throughout marrow	Heterogeneous: includes lineage-restricted (MALPs), bipotent (Adipoq^+^Osx^+^), and potentially multipotent (Adipoq^+^ SSPCs) subsets	Subset of Adipoq-lineage cells; Adipoq^+^ cells excluding mature adipocytes; encompasses MALPs, Adipoq^+^Osx^+^ cells, and Adipoq^+^ SSPCs; largely overlaps with CAR/LepR^+^ cells
Adipoq^+^ SSPCs (Hypothesized)	Hypothesized population of bona fide skeletal stem and progenitor cells that express *Adipoq* and possess self-renewal capacity and multipotent differentiation potential (e.g., osteoblasts, chondrocytes, adipocytes, and stromal cells).	Unknown	Unconfirmed multipotent differentiation potential (e.g., osteoblasts, chondrocytes, adipocytes, and stromal cells).	Hypothetical subset of Adipoq^+^ cells or Adipoq−lineage progenitors; not yet proven to exist
Adipoq−Lineage Progenitors	Ever expressed *Adipoq*; undifferentiated; lack Perilipin and lipid droplets	Perivascular, stromal, and endosteal niches	Heterogeneous: includes both lineage−restricted (MALPs) and bipotent (Adipoq^+^Osx^+^) progenitors	encompassing MALPs, Adipoq^+^Osx^+^ cells, Adipoq^+^ SSPCs, and their immature progeny; largely overlaps with CAR/LepR^+^ populations
MALPs (Marrow Adipogenic Lineage Precursors)	Highly expressing *Adipoq*, *Pparg*, *Cebpa*, *Apoe*, and *Lpl*; but lacking perilipin and lipid and osteogenic markers (*Sp7*, *Alpl*)	Perivascular niches and reticular stromal spaces throughout marrow	Lineage−restricted adipogenic under homeostasis in young mice; may acquire osteogenic potential with age or injury	Subset of Adipoq^+^ stromal cells or Adipoq−lineage progenitors; anatomically and functionally distinct from Adipoq^+^Osx^+^ cells; overlap largely with CAR cells and LepR^+^ cells; give rise to mature marrow adipocytes
Adipoq^+^Osx^+^ Cells	Co-expressing both adipogenic (*Adipoq*, *Cebpa*) and osteogenic (*Alpl*, *Sp7*) genes	Endosteal niche (predominantly)	Bipotent: adipo−osteoprogenitors under steady−state conditions	Subset of Adipoq^+^ stromal cells or Adipoq−lineage progenitors; anatomically and functionally distinct from MALPs
Mature Marrow Adipocytes	Perilipin^+^, Adipoq^+^, lipid−laden	Scattered throughout marrow; enriched in caudal vertebrae and aged marrow	Terminally differentiated; no further differentiation	Derived from MALPs and Adipoq^+^Osx^+^ cells; distinct from peripheral adipocytes

## 2 Identity of bone marrow Adipoq-lineage progenitors: adipo-progenitors, adipo-osteoprogenitors, or bona fide SSPCs

### 2.1 MALPs as authentic adipogenic precursors

Analysis of scRNA-seq data of *Col2-Cre-*targeted mesenchymal cells from 1- to 1.5-month-old mice, Zhong et al. identified a large cluster expressing *Adipoq*, *Pparg*, *Cebpa*, *Apoe*, and *Lpl* but lacking perilipin and lipid droplets (7). These cells, known as marrow adipogenic lineage precursors (MALPs), were initially proposed as committed adipocyte progenitors (7, 10). Fate mapping with *Adipoq-Cre* and/or *Adipoq-CreER* demonstrated that MALPs give rise to Perilipin^+^ lipid-laden mature adipocytes both *in vitro* and *in vivo*. Notably, in 1- and 3-month-old mice, Adipoq-lineage cells, predominantly MALPs at these ages, did not incorporate EdU, rarely contributed to osteoblasts, and failed to form CFU-F colonies *in vitro* (7, 10), positioning them as lineage-restricted adipo-progenitors for marrow adipogenesis.

Morphologically, MALPs form a striking three-dimensional network via long cellular processes, distributing throughout the bone marrow as reticular stromal cells or wrapping around blood vessels as pericytes (9–11). Ablation of Adipoq-lineage cells using *Adipoq-Cre; Rosa26-iDTR* mice caused severe vascular dilation, reduced endothelial cell numbers, and paradoxically induced *de novo* trabecular bone formation in the diaphysis. Since MALPs represent the largest subpopulation of Adipoq-lineage cells, these results suggest that MALPs actively suppress osteogenesis while maintaining marrow vasculature (7). However, because *Adipoq-Cre* targets the entire lineage, a MALP-specific contribution to this phenotype cannot be definitively determined.

### 2.2 Adipoq^+^Osx^+^ cells: bipotent adipo-osteoprogenitors

However, several lines of evidence suggest that Adipoq-lineage cells can acquire osteogenic potential with age and under specific conditions (5, 9, 11–14). Mukohira et al. found that although Adipoq^+^ stromal cells appear as early as one day after birth in mouse bone marrow, their contribution to *Alpl^+^* osteoblasts increases progressively from 4 to 24 weeks of age (12). Consistent with this observation, lineage tracing using *Adipoq-CreER^T2^* from Long group confirmed that Adipoq-lineage cells did not contribute to osteoblasts at 2 weeks (skeletal growth stage) but become an important source of endosteal osteoblasts from 4 weeks onwards (9, 13). Moreover, Adipoq-lineage cells give rise to most osteoblasts during bone regeneration from drill-hole injury (11), and expression of *Gsα^R201C^* in these cells stimulates trabecular bone formation (14). To further dissect this heterogeneity, Liao et al. recently performed scRNA-seq of sorted *tdTomato*^+^ cells from tamoxifen-injected 8-week-old *Adipoq-CreER^T2^; Ai9* mice and identified a smaller subset of cells co-expressing *Adipoq* and *Sp7* (encoding Osterix), termed Adipoq^+^Osx^+^ cells, which localize predominantly to the endosteal niche. These cells express both adipogenic (*Adipoq*, *Cebpa*) and osteogenic (*Alpl*, *Sp7*) genes, and their number is higher in young females than males, declining with age in both sexes (9). Using intersectional genetics with *Sp7-DreER* and *Adipoq-CreER^T2^*, Liao et al. demonstrated that under steady-state conditions, Adipoq^+^Osx^+^ cells give rise to osteoblasts on trabecular and endocortical surfaces as well as Perilipin^+^ marrow adipocytes in adult mice, establishing them as bipotent adipo-osteoprogenitors. Consistent with this concept, conditional deletion of *Sp7* with *Adipoq-Cre* reduced trabecular bone mass but, interestingly, did not affect cortical bone (9). This observation aligns with a previous report demonstrating that *Adipoq-Cre*-targeted cells contribute significantly to trabecular, but little to cortical, bone formation (14). Importantly, scRNA-seq of human bone marrow identified a subset of *ADIPOQ*^+^ stromal cells that co-express the niche markers *CXCL12* and *LEPR* along with both adipogenic and osteogenic genes, suggesting that marrow Adipoq-lineage cells include bipotent adipo-osteogenic progenitors in humans (15).

### 2.3 Relationship of Adipoq^+^ stromal cells to other SSPC populations

The identification of Adipoq^+^Osx^+^ cells as bipotent progenitors raises the question of how these cells relate to other SSPC populations. Notably, scRNA-seq and co-expression studies indicate that Adipoq^+^ stromal cells share expression of *Cxcl12*, *LepR*, with the CXCL12-abundant reticular (CAR) and leptin receptor-expressing (LepR^+^) cells, and Adipoq is also expressed in the majority of PDGFRβ^+^VCAM-1^+^ stromal cells (1, 9, 12), suggesting that Adipoq^+^ stromal cells are transcriptionally related to well-characterized SSPC populations. In terms of lineage relationship, most stromal cells targeted by *Adipoq-Cre* are CAR cells, though they represent only a subset of the broader CAR cell population. Similarly, *Adipoq-Cre* targets the majority of PDGFRβ^+^VCAM-1^+^ stromal cells and a fraction of marrow LepR^+^ SSPCs, but not periosteal LepR^+^ SSPCs. In humans, scRNA-seq and spatial proteomic imaging of femoral bone marrow has identified Adipo-MSCs that transcriptionally resemble mouse MALPs/Adipo-CAR cells, confirming the conservation of this population across species (16). Functionally, CAR cells largely overlap with LepR^+^ cells, and both are known to be major sources of CFU-Fs, and are broadly osteogenic and adipogenic (17, 18). However, as discussed above, Adipoq-lineage cells exhibit a more restricted fate under homeostatic conditions, although this restriction depends on age and anatomical location. Thus, while marker and spatial overlaps exist, CAR/LepR^+^ cells and Adipoq-lineage cells are not functionally equivalent. Nevertheless, while *Adipoq-Cre*-labeled cells from 1-month-old mice do not form CFU-Fs *in vitro (*7) and Lu et al. reported that almost all CFUs from adult *Adipoq-CreER^T2^*; *tdTomato* mice are derived from *tdTomato^-^* cells (13), both constitutive and inducible *Adipoq-Cre* label most CFU-Fs at 8–24 weeks of age (11). Consistent with these observations, a significant portion of endosteal Adipoq-lineage cells incorporate EdU by 27 weeks (9). Therefore, although Adipoq^+^ stromal cells in young mice appear largely restricted to the adipogenic lineage, the possibility that these cells contain bona fide multipotent SSPCs with self-renewal capacity (termed Adipoq^+^ SSPCs), particularly with age, cannot be definitively excluded.

## 3 Diverse functions of bone marrow Adipoq-lineage cells

### 3.1 Role of bone marrow Adipoq-lineage cells in osteoclastogenesis

**Figure 1:**
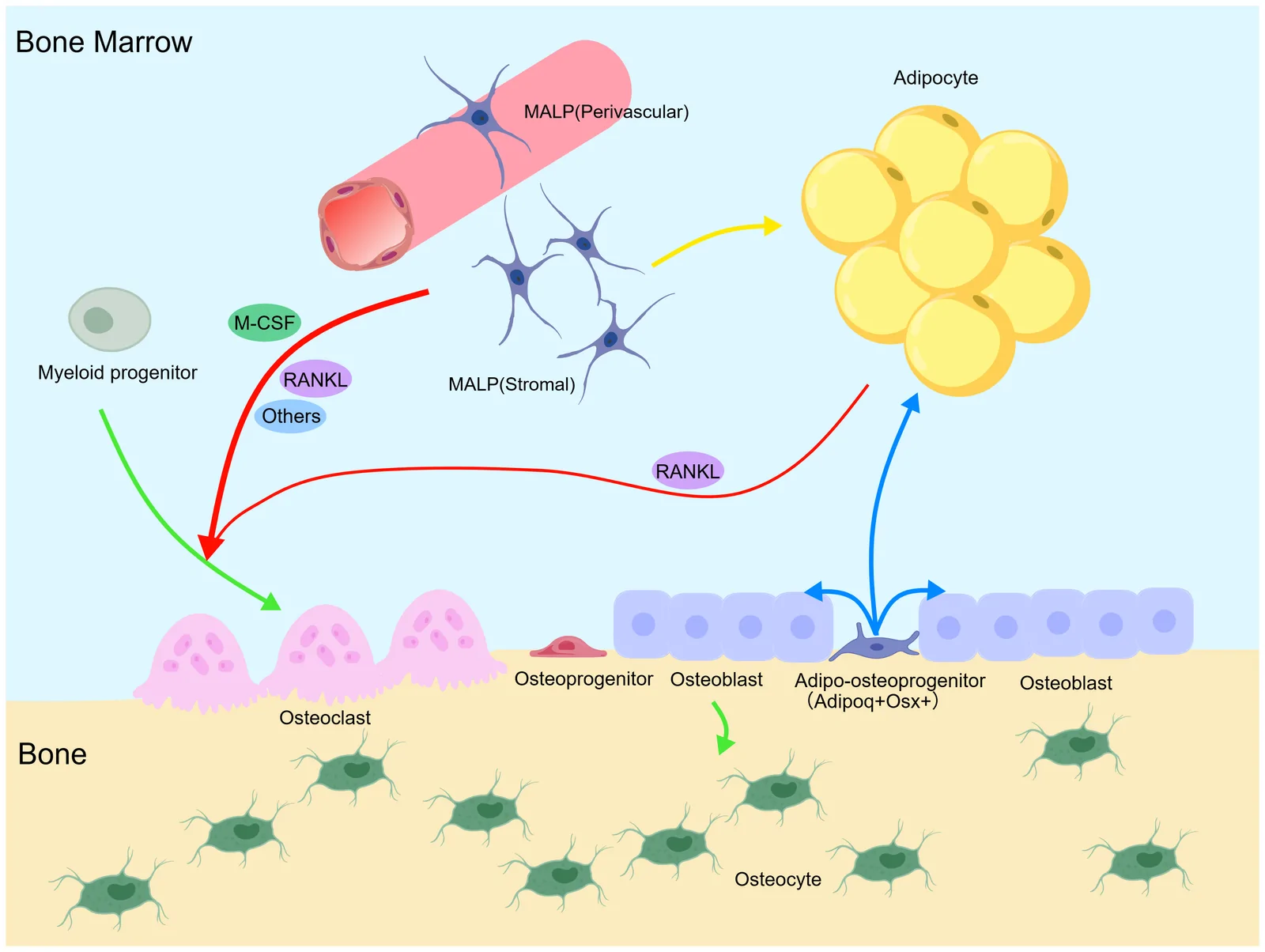
Bone marrow Adipoq-lineage cells (MALPs and mature adipocytes) promote osteoclastogenesis through secreted factors including M-CSF, RANKL, and additional mediators. Green arrows indicate the differentiation trajectory from myeloid progenitors to mature osteoclasts. Yellow and blue arrows denote lineage progression within Adipoq-lineage cells: yellow indicates unipotent differentiation of MALPs into mature adipocytes, while blue indicates bipotent differentiation of Adipoq^+^Osx^+^ cells into osteoblasts and adipocytes. Thick and thin red arrows denote pro-osteoclastogenic factors secreted by MALPs and mature adipocytes, respectively; the thicker arrow emphasizes that MALPs serve as the major source of RANKL compared with mature adipocytes. “Others” refers to additional pro-osteoclastogenic factors highly enriched in MALPs, such as IL-7, IL-34, CCL2, VCAM-1, and C3.

While marrow Adipoq-lineage cells contain adipo-osteoprogenitors with intrinsic osteogenic potential, they can also indirectly influence bone metabolism by acting on osteoclast-mediated bone resorption and osteoblast-mediated bone formation. Stepwise formation of mature functional osteoclasts from myeloid progenitors, a process known as osteoclastogenesis, is driven by two essential factors, including macrophage colony-stimulating factor (M-CSF) and receptor activator of nuclear factor kappa-B ligand (RANKL) (19–23). A paradigm shift has occurred with the recognition that Adipoq-lineage cells are the predominant source of both M-CSF and RANKL (**Figure 1**).

Using constitutive *Adipoq-Cre*, multiple studies demonstrated that conditional deletion of *Tnfsf11* (encoding RANKL) or *Csf1* (encoding M-CSF) in Adipoq-lineage cells leads to a marked increase in trabecular bone mass due to suppressed osteoclast formation, without affecting cortical bones, a finding partly explained by markedly reduced RANKL expression in the bone marrow but not in cortical bone (10, 24–26). Moreover, these mice are protected from ovariectomy-induced trabecular bone loss and LPS-induced calvarial bone loss, all attributed to suppressed osteoclastogenesis (10, 24–26). The subsequent use of an inducible *Adipoq-CreER* system confirmed that these effects are not due to developmental defects and that Adipoq-lineage cells continue to be the major RANKL source in adult mice: depletion of these cells in adult mice rapidly increases trabecular bone mass, and prevents and even restores osteoporotic bone loss caused by ovariectomy (13). Furthermore, several other cytokines and chemokines important for osteoclastogenesis are also strongly produced by Adipoq-lineage cells (10). scRNA-seq and *in situ* expression characterization have further revealed that MALPs express the highest levels of RANKL and M-CSF in the bone marrow, surpassing other mesenchymal lineage cells (10, 13, 24–26), suggesting that MALPs are the predominant source of these two factors. However, this cannot be formally proven with the current genetic approach, since the *Adipoq-Cre* or *Adipoq-CreER* tools do not distinguish between MALPs and other *Adipoq-Cre*-targeted cells. In addition to MALPs, mature bone marrow adipocytes, but not peripheral adipocytes, also express RANKL (27). Thus, Adipoq-lineage cells regulate osteoclastogenesis by producing M-CSF, RANKL, and other factors (**Figure 1**).

Despite the compelling evidence for Adipoq-lineage cells as key regulators of osteoclastogenesis, several lines of evidence indicate that these cells are not the exclusive source of osteoclastogenic cytokines. First, deletion of either *Tnfsf11* or *Csf1* in Adipoq-lineage cells did not affect cortical bones. Second, deletion of *Tnfsf11* in all mesenchymal cells using *Prrx1-Cre* causes significantly more severe osteopetrotic phenotypes than its deletion in Adipoq-lineage cells alone (26). Third, *Adipoq-Cre*-driven *Csf1* conditional knockout mice show an osteopetrotic phenotype in long bones, but their vertebrae are unaffected, despite similar frequencies of Adipoq-lineage progenitors in both locations (26). These phenotypic discrepancies strongly suggest that alternative cellular sources contribute substantially to RANKL and M-CSF production in bone. Indeed, under physiological conditions, osteoprogenitors positioned on the bone surface in the vicinity of osteoclasts express high levels of RANKL, a finding observed in both human and mouse skeletal tissues (28). In addition, osteocytes have been identified as a significant source of RANKL essential for bone remodeling and unloading-induced bone loss (29–31). Similarly, M-CSF can be derived from sources other than Adipoq-lineage cells. Previous studies indicate that osteocytes are an important source of M-CSF in the bone marrow (32). Nevertheless, despite the established functional importance of osteoblast/osteocyte-derived RANKL and M-CSF in osteoclastogenesis, scRNA-seq and some *in situ* hybridization studies reveal nearly undetectable mRNA expression of these cytokines in these cells (13). Thus, the relative contribution of Adipoq-lineage cells-derived versus other mesenchymal cell-derived RANKL and M-CSF to osteoclastogenesis in different skeletal sites remains unresolved.

### 3.2 Role of bone marrow Adipoq-lineage cells in regulating osteogenesis and marrow vasculature

**Figure 2:**
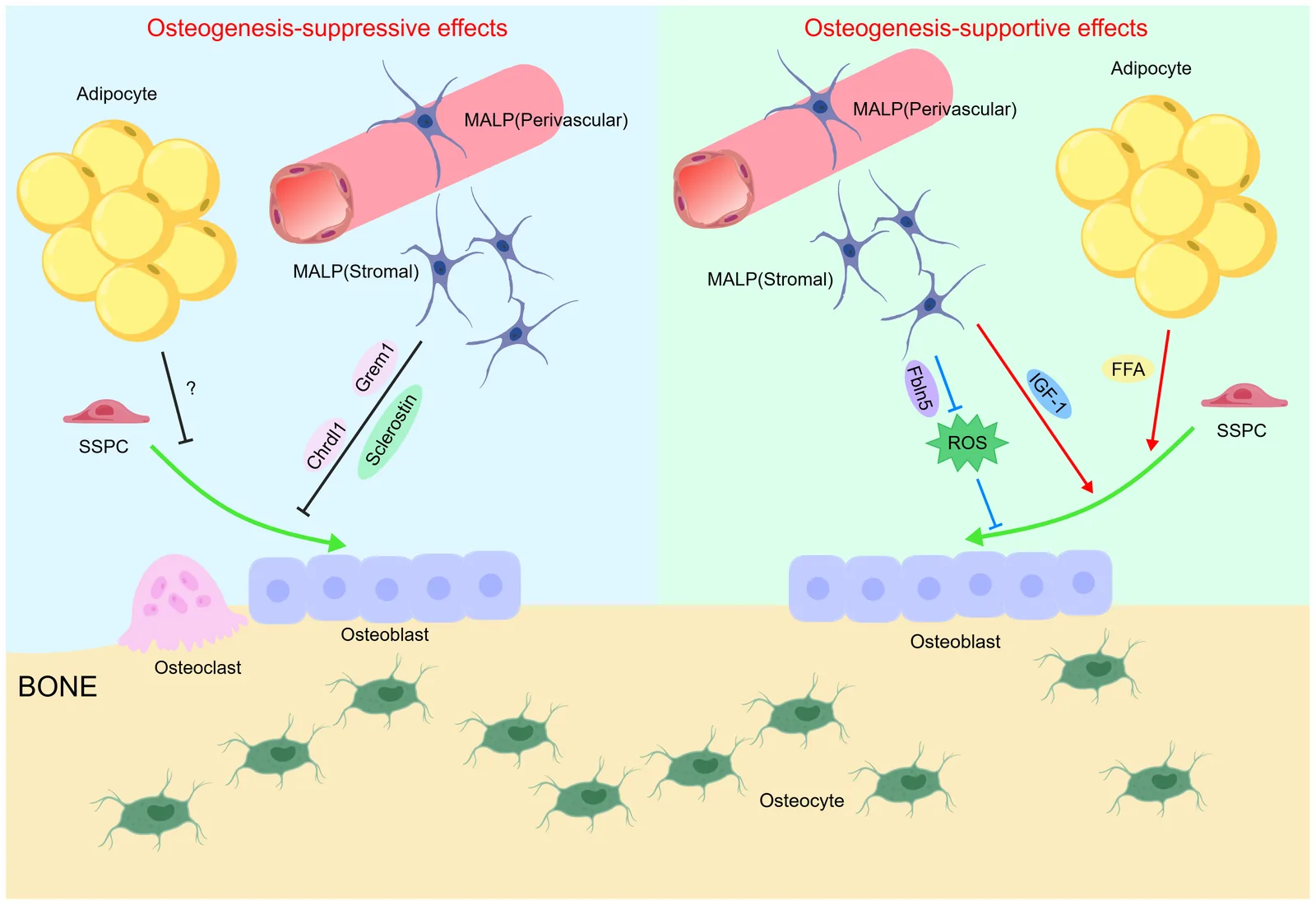
Bone marrow Adipoq-lineage cells indirectly regulate osteogenesis. Green arrows denote differentiation trajectories from SSPCs toward osteoblasts. Left panel summarizes the osteogenesis-suppressive effects of Adipoq-lineage cells (including MALPs and mature adipocytes). Black T-bars indicate that MALPs or adipocytes suppress osteogenic differentiation via the secreted factors shown in the diagram or via unknown factors (indicated by a question mark). Right panel illustrates the context-dependent osteogenesis-supportive effects of these cells. Red arrows indicate context-dependent activation of osteogenic differentiation by the secreted factors (IGF 1) or free fatty acids (FFA) shown in the diagram; blue T-bars denote negative regulation of ROS by MALPs via Fbln5, as well as the inhibitory effect of ROS on osteogenesis.

Beyond their role in osteoclastogenesis, marrow Adipoq-lineage cells indirectly influence osteogenesis and angiogenesis. Genetic ablation studies have established that these cells are essential for marrow vascular maintenance and regeneration. However, despite the tight coupling between osteogenesis and Type H (CD31^+^Emcn^+^) vessel formation, the net effect of Adipoq-lineage cells on osteogenesis itself has been inconsistent across studies, likely reflecting a balance between pro-osteogenic and anti-osteogenic secreted factors. The production of these factors is itself subject to cell-intrinsic regulation, which is discussed separately in Section 4. To clarify this complexity, we organize the evidence as follows. First, we examine systemic regulation by peripheral adipose tissue. We then turn to local regulation by marrow-resident Adipoq-lineage cells, distinguishing osteogenesis-suppressive mechanisms from supportive ones, and finally address context-dependent metabolic support during energy stress (**Figure 2**).

#### 3.2.1 Systemic suppression of osteogenesis by peripheral adipose tissues

Congenitally lipodystrophic (*Adipoq-Cre; DTA*) mice exhibited a near complete absence of peripheral white and brown adipose tissues (WAT and BAT), yet marrow adipocytes remained unaltered (33). These mice developed profound and rapid osteosclerosis, with a dramatic expansion of trabecular bone mass, due to markedly stimulated osteogenesis rather than reduced resorption. Importantly, this high-bone-mass phenotype was largely rescued by subcutaneous transplantation of wild-type WAT or BAT, but not by adipose tissues derived from mice lacking both adiponectin and leptin. These findings demonstrate that peripheral adipose tissues suppress bone formation, at least in part, through the combined actions of adiponectin and leptin (33).

#### 3.2.2 Local suppression of osteogenesis by marrow Adipoq-lineage cells

The congenital model established that peripheral fat exerts systemic inhibitory effects on bone formation. However, it left unresolved whether marrow-resident Adipoq-lineage cells have similar functions. To address this, two independent studies employed inducible cell ablation strategies (7, 34). Ablation of Adipoq-lineage cells in 1-month-old mice using *Adipoq-Cre; Rosa26-iDTR* (which targets both peripheral adipocytes and marrow Adipoq-lineage cells) caused severe vascular dilation, reduced endothelial cell numbers, and paradoxically induced *de novo* trabecular bone formation in the diaphysis after two weeks of diphtheria toxin-induced ablation (7). Unlike the congenital model, these effects could not be rescued by transplantation of wild-type subcutaneous fat, indicating that marrow Adipoq-lineage cells actively suppress osteogenesis while maintaining marrow vasculature in young mice (7). Consistent with this finding, Zou et al. demonstrated that specifically ablating Adipoq-lineage cells in 2-month-old mice using the same strategy rapidly accelerated osteogenesis within 4 days after ablation and led to massive bone gain within 10 days (34). This effect could not be rescued by transplantation of wild-type WAT. Instead, it was mediated by elimination of the BMPR inhibitors Grem1 and Chrdl1, resulting in BMPR activation and proliferation of pre-osteoblasts (34, 35). Forced re-expression of these two inhibitors via nanoparticles reversed these phenotypes (35). Moreover, inducible ablation of Adipoq-lineage cells protected against ovariectomy-induced and age-related bone loss (34). These results demonstrate that Adipoq-lineage cells suppress osteogenesis by secreting BMPR inhibitors in adult mice. Moreover, conditional deletion of *Sost* (encoding sclerostin) in Adipoq-lineage cells increased trabecular bone mass, promoted CFU-F and CFU-OB formation, and enhanced osteogenic differentiation of bone marrow stromal cells without affecting adipogenesis and osteoclastogenesis (36). These results indicate that in addition to secreting BMPR inhibitors, Adipoq-lineage cells also produce sclerostin to locally suppress bone formation. Because all above functional studies have used *Adipoq-Cre* or *Adipoq-CreER^T^*² drivers that target the entire lineage, it is not possible to definitively assign the osteogenesis-suppressive function to any single subset of Adipoq-lineage cells. However, the rapid bone gain after cell ablation (within days) and the fact that marrow adipocytes are rare in young mice indirectly suggest that MALPs are the primary source of the secreted osteogenic suppressors (Grem1, Chrdl1, sclerostin). However, this remains an inference rather than a proven conclusion. Moreover, the above studies did not rule out a role for mature marrow adipocytes in suppressing bone formation. Indeed, selective depletion of mature marrow adipocytes achieved through a novel intersectional dual-recombinase strategy led to increased cortical and trabecular bone mass, primarily due to enhanced endosteal bone formation (37). This targeted depletion also protected mice from cortical bone loss induced by caloric restriction or ovariectomy and promoted bone healing after fracture. Thus, mature marrow adipocytes also play a suppressive role in regulating osteogenesis.

#### 3.2.3 Local promotion of osteogenesis and vascular support by Adipoq-lineage cells via Fbln5-mediated regulation of redox homeostasis

Of note, while ablation of Adipoq-lineage cells in young adult mice (1–2 months old) increases bone mass, ablation of the same cell lineage during late gestation leads to bone loss, suggesting that the role of Adipoq-lineage cells in osteogenesis is highly dependent on developmental stage. Indeed, Su et al. found this discrepancy is likely caused by distinct cellular origins of Adipoq^+^ cells in perinatal versus adult bone marrow (38). Bone marrow Adipoq^+^ cells express fibulin-5 (Fbln5), an extracellular matrix scaffolding protein that interacts with superoxide dismutase 3 (SOD3) to regulate ROS levels (38). Genetic ablation of Adipoq-lineage cells during late gestation downregulated Fbln5 expression, elevated ROS stress and cellular senescence in the bone marrow of young mice, and consequently reduced type H vessels as well as trabecular and cortical bone mass, phenotypes recapitulated by prenatal dexamethasone exposure (PDE). Conversely, targeted overexpression of Fbln5 in Adipoq-lineage cells via AAV at 2 weeks of age rescued the PDE-induced ROS accumulation, cellular senescence, type H vessel loss, and bone loss, without affecting these parameters in wild type controls. Notably, PDE did not increase Perilipin^+^ mature adipocytes, indicating that the protective function is conferred by MALPs rather than by mature adipocytes. These findings indicate that Fbln5 is a key mediator of the protective function of Adipoq-lineage cells (predominantly MALPs) under stress conditions. To further confirm the critical role of Fbln5 in mediating PDE-induced bone loss, it will be important to examine whether Adipoq-lineage-specific deletion of *Fbln5* recapitulates the phenotypes observed in the above ablation studies. Moreover, whether this protective function is restricted to the perinatal/early postnatal period remains to be determined.

#### 3.2.4 Local promotion of endocortical bone formation by Adipoq-lineage cell-derived IGF-1

IGF-1 is highly enriched in Adipoq^+^ CAR cells (39). Conditional deletion of *Igf1* using *Adipoq-Cre* reduces cortical bone thickness specifically in 12-week-old female mice due to suppressed endosteal bone formation, without affecting trabecular bone, marrow adiposity, or hematopoiesis (39). This study highlights a site-specific, sex-dependent paracrine role for IGF-1 from Adipoq-lineage cells (predominantly MALPs), though potential redundancy with other IGF-1 sources warrant further investigation.

#### 3.2.5 Context-dependent metabolic support: marrow adipocyte lipolysis fuels osteogenesis under energy stress

Using a marrow adipocyte-specific *Pnpla2* knockout model, Li et al. demonstrated that impaired adipocyte lipolysis does not affect bone mass under ad libitum feeding but causes significant trabecular bone loss under caloric restriction, cold exposure, and during bone regeneration in a sex-dependent manner (40). These findings establish that adipocyte lipolysis fuels osteoblast activity when systemic energy is limited, revealing a distinct energy-supplying function of marrow adipocytes that complements their previously described paracrine regulatory roles.

Collectively, these studies establish that Adipoq-lineage cells regulate osteogenesis through multiple inhibitory or stimulatory mechanisms. The net effect on bone mass thus depends on developmental stage, skeletal site, sex, and metabolic context. A seeming paradox arises because the same Adipoq-lineage both suppresses osteogenesis (via paracrine inhibitors) and directly contributes to osteoblasts (via Adipoq^+^Osx^+^ cell differentiation). This paradox is resolved by recognizing that suppression and direct contribution occur at different timescales and magnitudes. Paracrine suppression derived from Adipoq-lineage cells is rapid (within days) and quantitatively dominant under homeostasis, and its loss may derepress osteogenic potential of non-Adipoq-lineage osteoprogenitors, driving a burst of bone formation that overrides any deficit from losing the slower, modest contribution of Adipoq^+^Osx^+^ cells. Thus, *Adipoq-Cre* ablation experiments reveal that the net effect of removing the entire lineage is dominated by loss of suppression. Distinguishing the specific roles of MALPs, Adipoq^+^Osx^+^ cells, and mature adipocytes will require more refined genetic tools that enable subset-specific targeting.

### 3.3 Role of bone marrow Adipoq-lineage cells in hematopoiesis

#### 3.3.1 Cytokine production by Adipoq-lineage cells

**Figure 3:**
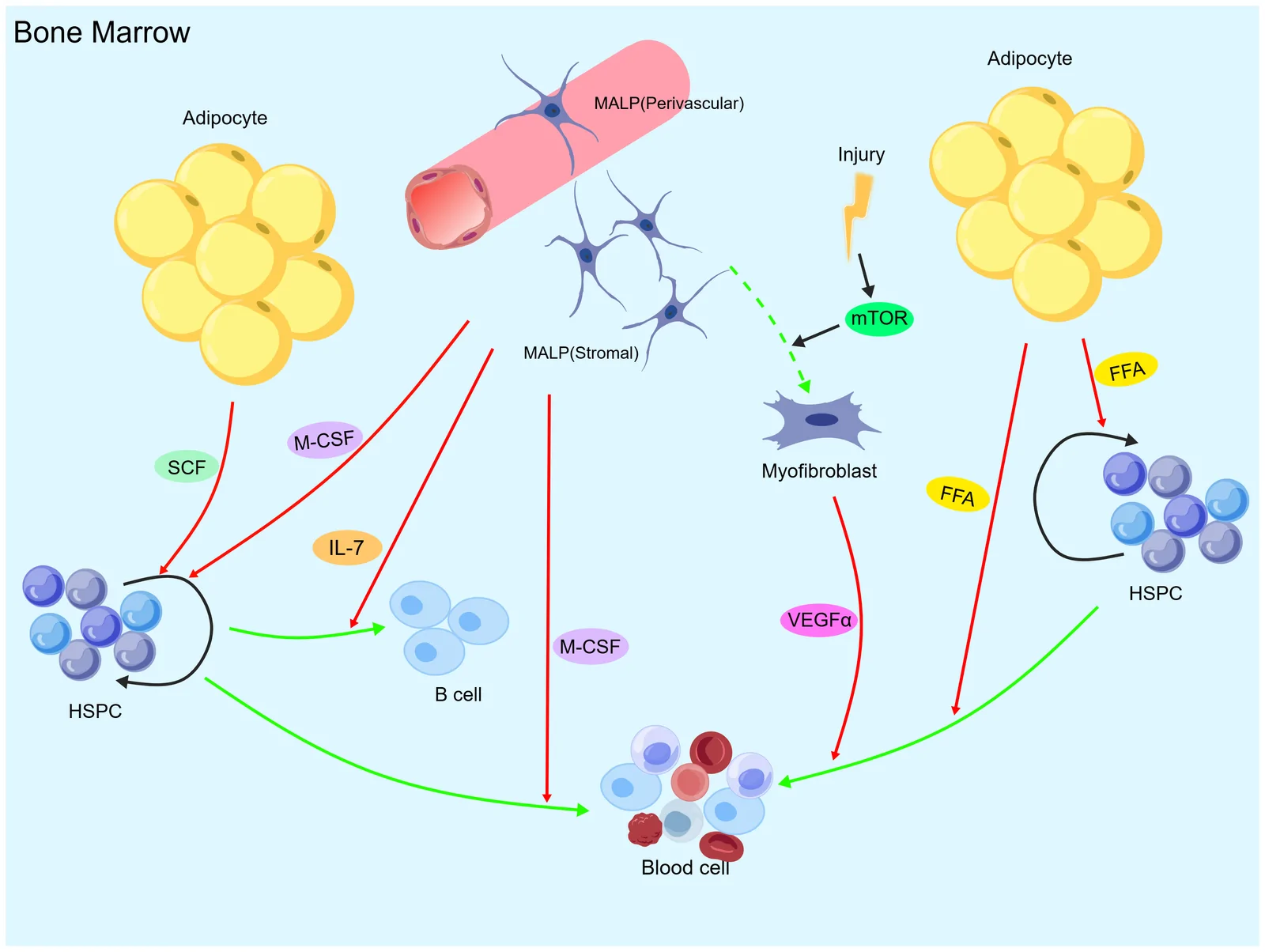
Adipoq-lineage cells play critical roles in supporting hematopoiesis. Circular black arrows indicate proliferation of hematopoietic stem/progenitor cells (HSPCs), while green arrows denote differentiation trajectories from HSPCs toward B cells and other mature blood lineages. Red arrows indicate context-dependent activation of these pathways by the secreted factors or free fatty acids (FFA) shown in the diagram, and the dashed green arrow denotes the transition of MALPs (marrow adipogenic lineage precursors) into myofibroblasts.

Adipoq-lineage cells have emerged as essential components of the hematopoietic niche, primarily through the production of key cytokines such as SCF, M-CSF, and IL-7 (**Figure 3**). However, recent studies reveal that mature adipocytes and their precursors contribute to distinct aspects of hematopoietic support. Specifically, Li et al. found that selective depletion of marrow mature adipocytes in mice reduced HSPC numbers in marrow adipocyte-abundant sites (37). Furthermore, using an inducible *Adipoq-CreER* system to delete *Kitl* (encoding SCF) postnatally, Zhou et al. demonstrated that SCF derived from Adipoq-lineage cells is dispensable for steady-state hematopoiesis in long bones (where adipocytes are scarce) but is critical for hematopoietic stem cell (HSC) maintenance in caudal vertebrae (where adipocytes are abundant) (41), suggesting that Adipoq-lineage-derived SCF, particularly from marrow adipocytes, critically promotes HSPC maintenance. More importantly, after irradiation or 5-FU-induced myeloablation, SCF from mature adipocytes becomes indispensable for the regeneration of HSCs, LSK (Lin^-^Sca-1^+^c-Kit^+^) cells, and overall bone marrow cellularity, and its loss significantly impairs mouse survival (41). Notably, using a constitutive *Adipoq-Cre*, Zhang et al. showed that *Kitl* deletion leads to macrocytic anemia, reduced LSK cells, and diminished myeloid progenitors under steady-state conditions, and impairs responses to metabolic stresses including high-fat diet, β3-adrenergic activation, and aging (42). This suggests that SCF derived from the broader Adipoq-lineage cells (including MALPs) contributes to basal hematopoiesis. Thus, Adipoq-lineage cell-derived SCF is critical for hematopoietic regeneration and contributes to local steady-state hematopoiesis in an anatomically restricted manner.

Regarding M-CSF, two independent studies using scRNA-seq and conditional knockout approaches conclusively identify MALPs as the predominant cellular source of M-CSF (encoded by *Csf1*) in the bone marrow, whereas mature, Perilipin1^+^ lipid-laden adipocytes produce negligible levels. Conditional deletion of *Csf1* in these Adipoq-lineage cells reduces bone marrow macrophages, HSPCs (especially LK and LSK cells), and impairs hematopoietic recovery after injury, underscoring the unique role of this population (25, 26). In addition to SCF and M-CSF, Adipoq-lineage cells support B lymphopoiesis through IL-7 production. Mukohira et al. showed that MALPs, but not mature adipocytes, produce IL-7, and conditional deletion of *Il7* using *Adipoq-Cre* severely impaired B cell development in the bone marrow. These results establish that MALPs are an important source of IL-7 required for B lymphopoiesis (12).

Collectively, these studies demonstrate that Adipoq-lineage cells are critical niche cells that produce SCF, M-CSF, and IL-7, and thereby support steady-state hematopoietic homeostasis and/or regeneration (**Figure 3**). Importantly, proteomic imaging of human bone marrow revealed that primitive SPINK2^+^ HSPCs are significantly closer to adipocytes than to any other microanatomical structure, providing direct human evidence for the adipocyte-HSPC interaction (43).

#### 3.3.2 Myofibroblast conversion of MALPs in marrow repair after injury

Beyond cytokine production, MALPs also exhibit remarkable phenotypic plasticity in response to injury. Zhong et al. (2022) and Wang et al. (2025) demonstrated that MALPs undergo a striking phenotypic switch upon bone marrow injury induced by radiation or chemotherapy (5-FU) (44, 45). scRNA-seq revealed that, shortly after injury, MALPs transiently enter the cell cycle, expand rapidly, and acquire a myofibroblast phenotype, driven partly by activation of mTOR signaling. This myofibroblastic conversion is essential for subsequent bone marrow recovery, since ablation of Adipoq-lineage cells (including MALPs) using *Adipoq-Cre*-driven diphtheria toxin receptor (DTR) severely impaired the restoration of hematopoietic stem/progenitor cells, mature blood lineages, and bone marrow vasculature. Mechanistically, MALP-derived VEGFα was identified as one of the key paracrine factors mediating revascularization and hematopoiesis recovery after injury (**Figure 3**), though it only partially accounts for the full reparative effect. Notably, aging did not induce the same myofibroblast conversion, indicating that this plasticity is injury-specific rather than a general consequence of cellular senescence.

#### 3.3.3 Metabolic support of emergency hematopoiesis by marrow adipocytes after myocardial infarction

In addition to cytokine-mediated niche functions, marrow Adipoq-lineage cells, particularly bone marrow adipocytes, provide metabolic fuel to HSPCs during stress (46). Zhang et al. demonstrated that after myocardial infarction (MI), emergency hematopoiesis is fueled by fatty acid oxidation (FAO) in HSPCs (46). Granulocyte-monocyte progenitors (GMPs) from post-MI mice exhibited increased oxidative phosphorylation, FAO gene signatures, higher mitochondrial content, and elevated lipid uptake. In humans, HSPC lipid content also increased after acute MI. Conditional deletion of *Cpt1a* (encoding a rate-limiting enzyme for FAO) in hematopoietic cells abrogated post-MI HSPC proliferation, myeloid expansion, and monocyte/neutrophil recruitment to the heart. Importantly, inhibiting lipolysis in Adipoq-lineage cells via *Adipoq-CreER^T2^* -mediated deletion of *Atgl* or via inducible depletion of these cells by local diphtheria toxin injection in *Adipoq-CreER^T2^; iDTR* mice reduced post-MI HSPC proliferation and myeloid output. Of note, mature marrow adipocytes shrank rapidly after MI in both mice and humans, indicating that MI induces adipocyte lipolysis. Collectively, these findings establish that Adipoq-lineage cells, particularly marrow adipocytes, act as local energy stores, releasing fatty acids through lipolysis to support the increased metabolic demands of emergency hematopoiesis following ischemic injury (**Figure 3**). However, whether MALPs also contribute to this process remains unclear. Moreover, whether Adipoq-lineage cells regulate basal hematopoiesis by providing metabolic support has yet to be addressed.

### 3.4 Implications of Adipoq-lineage cells in human skeletal diseases

The growing understanding of pathophysiological roles of Adipoq-lineage cell in mice raises the question of whether these cells contribute to human skeletal pathologies, such as osteoporosis, osteoarthritis, bone marrow fibrosis, bone tumor microenvironment, diabetic bone disease, aging-related marrow adiposity, and fibrous dysplasia (FD). Notably, scRNA-seq has identified ADIPOQ^+^ stromal cells in human bone marrow, and these cells also express high levels of *M-CSF* and *SHN3*, consistent with findings from murine studies (25, 47). Moreover, in patients with B-cell acute lymphoblastic leukemia (B-ALL), adipogenic progenitors, which transcriptionally resemble Adipoq+ stromal cells, show a significant shift toward the adipogenic lineage (48). In mouse models, conditional expression of *Gsa^R201C^* in Adipoq-lineage cells recapitulates key features of human FD, including lytic lesions in cortical and trabecular bones (14). However, direct functional evidence linking human ADIPOQ^+^ stromal cells to skeletal diseases is currently lacking. Since most mechanistic insights into Adipoq-lineage cells are derived from mouse models, extrapolation of these finding to human pathology requires caution.

## 4 Regulatory mechanisms of Adipoq-lineage cells

While Section 3 focused on the functional outputs of Adipoq-lineage cells (i.e., the secreted factors through which they regulate osteoclastogenesis, osteogenesis, and hematopoiesis), this section addresses the upstream cell-intrinsic regulators that control their fate, functional state, or secretory phenotype. These regulators are summarized in **Table 2**.

**Table 2:** Key intrinsic regulators expressed in Adipoq-lineage cells.

Regulator	Source	Category	Primary Function(s)	Mechanism	Reference
SHN3	Adipoq-lineage progenitors	Transcriptional factor	Cell-autonomously restrains osteogenic fate; promotes adipogenesis	Unknown whether directly binds Runx2/PPARγ or regulates paracrine factors; deletion shifts progenitor fate toward osteogenesis *in vivo*	Li et al., 2024 (47)
FOXO1	Perivascular MALPs	Transcriptional factor	Preserves pericyte identity by preventing pericyte-to-myofibroblast conversion; supports type H vessel formation; maintains bone mass	Suppresses mTOR signaling; downstream targets unknown; deletion leads to pericyte-to-myofibroblast conversion, loss of type H vessels, and trabecular bone loss	Cheng et al., 2024 (49)
ESRRA	Adipoq-lineage cells (mainly marrow and peripheral adipocytes)	Nuclear receptor	Oppositely controls leptin and SPP1 secretion; regulates bone-fat-vessel axis	Directly activates Leptin promoter; represses Spp1 by interfering with E2/ESR1 signaling; deletion lowers leptin (relieves its suppression of BMSC osteogenesis) and increases SPP1 (promotes type H vessel formation)	Huang et al., 2024 (50)
Kindlin-2	Adipoq-lineage cells	Focal adhesion protein	Negatively regulates bone mass by promoting FABP4 expression	Binds FAS and prevents its degradation, activating PPARγ and increasing FABP4; Secreted FABP4 inhibits pancreatic β-cells, lowering insulin, and thereby reducing bone formation	Tang et al., 2023 (51)
DDR2	Adipoq-lineage cells (mainly marrow and peripheral adipocytes)	Receptor tyrosine kinase	Negatively regulates bone mass; suppresses lipolysis	Suppresses Adcy5/cAMP-PKA signaling; its deletion upregulates Adcy5, stimulating lipolysis to release FFAs, which are taken up by osteoblast progenitors via Ffar4 to boosts oxidative phosphorylation and osteogenesis	Yang et al., 2022 (52)

### 4.1 Schnurri-3 cell-autonomously restrains osteogenic fate of marrow Adipoq-lineage progenitors

scRNA-seq profiling revealed that *Shn3* (encoding SHN3) is highly expressed in Adipoq-lineage progenitors. Conditional deletion of *Shn3* using *Adipoq-Cre* mice led to a marked increase in trabecular bone mass and bone formation rate, without affecting cortical bone, systemic metabolism or peripheral adipose tissue function (47). *Shn3* deficiency shifts fate of these progenitors toward osteogenesis at the expense of adipogenesis, while secondarily elevating osteoclast numbers. *Shn3* ablation also promotes fracture healing. Thus, SHN3 is a key intrinsic regulator that restricts the osteogenic potential of Adipoq-lineage progenitors. However, whether SHN3 regulates paracrine factors or directly binds osteogenic or adipogenic transcription factors such as Runx2/PPARγ, and whether these effects are sex-dependent, remain to be determined. In addition, *Shn3* is also expressed in other cell types, and therefore it will be necessary to develop Adipoq-lineage-specific targeting strategies to treat skeletal diseases.

### 4.2 FOXO1 in perivascular MALPs positively regulates type H vessels and osteogenesis

FOXO1 expression in perivascular MALPs is essential for type H vessel formation, which couple angiogenesis with osteogenesis (49). Mechanistically, FOXO1 suppresses mTOR signaling in these pericytes, thereby preventing their myofibroblastic transformation and preserving pericyte identity and function. Age-related FOXO1 downregulation leads to pericyte-to-myofibroblast conversion, loss of type H vessels, and trabecular bone loss. Pharmacological inhibition of FOXO1 aggravated type H vessel degeneration and bone loss, whereas mTOR inhibitor rapamycin rescued these phenotypes, highlighting the FOXO1-mTOR axis as a critical regulator of vascular support in bone. However, it is still unknown whether the FOXO1-mTOR axis in MALPs regulates type H vessels through secreted factors or via cell-cell contact, and what the identities of such factors might be. Notably, MALPs also undergo myofibroblastic conversion upon bone marrow injury induced by radiation or chemotherapy (5 FU), driven partly by mTOR activation (44, 45). Intriguingly, this conversion is essential for subsequent recovery of bone marrow vasculature. Thus, a key unresolved question is why MALP to myofibroblast transition is essential for marrow vasculature recovery after injury but detrimental to type H vessel maintenance under homeostatic conditions. Moreover, whether loss of FOXO1 in pericytes mimics the injury response has yet to be determined.

### 4.3 ESRRA in Adipoq-lineage cells regulates osteogenesis of bone marrow stromal cells

Huang et al. demonstrated that the orphan nuclear receptor ESRRA oppositely controls leptin and osteopontin (SPP1) secretion from Adipoq-lineage cells (50). They used *Adipoq-Cre*- to ablate *Esrra* in Adipoq-lineage cells and showed that ESRRA deficiency protects against bone loss and excessive marrow adiposity in both diet-induced obesity and ovariectomy-induced osteoporosis (50). Their mechanistic study revealed that ESRRA directly binds the *Leptin* promoter to activate its expression, but represses *Spp1* transcription by interfering with E2/ESR1 signaling. Accordingly, ESRRA ablation reduces leptin and increases SPP1 secretion from bone marrow and visceral adipocytes. This shift in secreted factors ultimately induces bone marrow stromal cells toward osteogenesis and promotes type H vessel formation. Notably, ESRRA deletion does not affect the intrinsic differentiation capacity of Adipoq-lineage progenitors *in vitro*. Thus, Adipoq-lineage cells, via ESRRA-controlled leptin and SPP1, function as key regulators of the bone-fat-vessel axis. However, the relative contribution of marrow Adipoq-lineage cells versus peripheral adipocytes to circulating SPP1 and leptin remains unresolved.

### 4.4 Kindlin-2 in Adipoq-lineage cells indirectly restrains osteogenesis by promoting FABP4 expression

Kindlin-2, a focal adhesion protein highly expressed in adipocytes, has been shown to negatively regulate bone mass (51). Conditional deletion of *Kindlin-2* using *Adipoq-Cre* increases trabecular and cortical bone mass, improves bone mechanical properties, and enhances osteoblast formation without affecting osteoclastogenesis. Mechanistically, Kindlin-2 binds to fatty acid synthase (FAS) and prevents its ubiquitin-proteasomal degradation. Accordingly, Kindlin-2 loss accelerates FAS degradation, reducing PPARγ activation and downstream FABP4 expression in Adipoq-lineage cells. Lower circulating FABP4 relieves inhibition of pancreatic β-cells, increasing insulin secretion and insulin signaling in bone, thereby promoting osteogenesis. Pharmacological FAS inhibition with C75 or AAV-mediated Kindlin-2 knockdown recapitulates the high-bone-mass phenotype. Of note, it is still unclear whether Kindlin-2 deficiency also causes cell fate switching of Adipoq-lineage progenitors. Additionally, the direct molecular target of FABP4 on pancreatic β-cells is unidentified, and whether the high-bone-mass phenotype fully depends on elevated insulin signaling requires validation using β-cell-specific deletion of insulin receptor.

### 4.5 DDR2 in Adipoq-lineage cells negatively regulates bone mass by suppressing lipolysis

Discoidin domain receptor 2 (DDR2), a collagen-activated receptor tyrosine kinase, has been shown to mediate crosstalk between adipocytes and bone (52). Adipoq-lineage-specific *Ddr2* knockout mice are protected from high-fat diet-induced fat gain and exhibit significantly increased trabecular and cortical bone mass with enhanced mechanical properties (52). Mechanistically, DDR2 deficiency in these cells upregulates adenylate cyclase 5 (Adcy5), elevating cAMP-PKA signaling, which stimulates lipolysis and reduces bone marrow adiposity. Released free fatty acids (FFAs) are taken up by nearby osteoblasts via fatty acid transporters such as Ffar4, boosting mitochondrial oxidative phosphorylation and osteoblast differentiation. Although osteoclast numbers are also elevated, net bone mass increases due to dominant osteoblast stimulation. These effects are independent of systemic metabolic changes, and pharmacological inhibition of Adcy5 with SQ22536 abolishes the high-bone-mass phenotype. Despite these findings, several points remain to be clarified. DDR2 deficiency in Adipoq-lineage cells is associated with upregulated *Adcy5*, yet how DDR2 regulates *Adcy5* expression remains unexplored. The increased *Adcy5* mRNA may be an indirect consequence of altered metabolic states. In addition, activating *DDR2* mutations cause Warburg-Cinotti syndrome manifested by lipodystrophy and acro-osteolysis, indicating that DDR2 function is context-dependent and may vary across different adipose depots.

## 5 Conclusions and future directions

The studies reviewed here reveal bone marrow Adipoq-lineage cells as versatile regulators of bone and marrow microenvironment, by supporting hematopoiesis and vascular integrity, restraining bone formation under homeostasis, yet mobilizing for repair after injury. This functional flexibility reflects three principles, including anatomical heterogeneity within the lineage, context-dependent reprogramming, and functional partitioning between precursor and mature adipocyte states.

### 5.1 An integrated model of Adipoq-lineage heterogeneity

The relationship between MALPs and Adipoq^+^Osx^+^ cells has been a central unresolved question. The evidence now supports a hierarchical model in which perivascular/stromal Adipoq^+^ cells (mainly MALPs) are predominantly adipogenic under homeostasis (12, 14), whereas endosteal Adipoq^+^Osx^+^ cells function as bipotent adipo-osteoprogenitors, as definitively demonstrated by intersectional genetic fate mapping. This framework reconciles earlier lineage-restricted findings with subsequent bipotent observations by treating these populations as anatomically segregated states rather than contradictory identities. Consistent with this model, Adipoq^+^ stromal cells contribute to osteoblasts under steady-state conditions in adult bone marrow (11, 12, 14), and this osteogenic capacity is amplified by injury (11) or specific genetic perturbations such as *Shn3* loss or *Gsα^R201C^* overexpression (14, 47). However, because current *Adipoq-Cre* tools label the entire Adipoq-lineage and cannot distinguish contributions from different subsets, the relative contribution of Adipoq^+^Osx^+^ cells versus other Adipoq^+^ stromal cells to this osteogenic pool remains unresolved.

Another key unanswered question is whether Adipoq expression indicates lineage commitment or transient multipotency. Resolving this will require clonal lineage tracing and temporal single cell transcriptomics to map dynamic Adipoq expression during cell fate decisions.

### 5.2 Context-dependent functional reprogramming

The net effect of Adipoq-lineage cells on bone and hematopoiesis is fine-tuned by developmental stage, metabolic state, and injury status. Fbln5 expressed by Adipoq-lineage cells interacts with SOD3 to suppress oxidative stress and establish the vascular and osteogenic niche, a function that becomes evident when these cells are ablated before birth and analyzed in early postnatal life. Under adult homeostasis, MALPs restrain excessive bone formation via BMPR inhibitors and sclerostin. With aging, FOXO1 downregulation drives pericyte-to-myofibroblast conversion, progressively uncoupling angiogenesis from osteogenesis. During energy deficit or myocardial infarction, marrow adipocytes switch to lipolysis, providing fatty acids to fuel osteoblasts and HSPCs. After myeloablation, MALPs undergo mTOR-driven myofibroblast conversion and secrete VEGFα to drive revascularization. These context-dependent functional reprogramming imply that therapeutic timing is as critical as target selection.

### 5.3 Therapeutic translation: from fate reprogramming to secretory modulation

Three of the five intracellular regulators (namely ESRRA, Kindlin-2, and DDR2), act primarily by modulating the secretory phenotype of Adipoq-lineage cells, whereas SHN3 and FOXO1 function mainly through intrinsic control of progenitor fate and functional state. This suggests that therapeutic strategies should move beyond reprogramming cell fate and also target the secretory phenotype of Adipoq-lineage cells.

Three challenges must be addressed before translating findings from Adipoq-lineage cells into feasible therapeutic strategy. First, Adipoq-lineage cells secrete both beneficial and harmful factors, and therefore approaches for factor-specific modulation rather than global activation or ablation of this lineage needs to be developed. Second, the secretory profile of Adipoq-lineage cells is context-dependent, therefore necessitating real-time biomarkers to guide intervention timing. Finally, factors secreted by these cells act in confined microanatomical spaces. Accordingly, local delivery strategies such as biomaterial scaffolds or bone-homing AAV vectors are highly demanded.

Future studies are surely warranted to focus on the following priorities: mapping the complete secretome of MALPs versus mature adipocytes across contexts to identify druggable nodes that can selectively enhance osteogenic or hematopoietic support without disrupting vascular maintenance; engineering factor-specific interventions (e.g., anti-Grem1/Chrdl1 antibodies) and test them in disease models; and validating whether the secretory programs observed in mouse models, are conserved in human bone marrow.
